# Effect of Tool Geometry Parameters on the Formability of a Camera Cover in the Deep Drawing Process

**DOI:** 10.3390/ma14143993

**Published:** 2021-07-16

**Authors:** Thanh Trung Do, Pham Son Minh, Nhan Le

**Affiliations:** 1 Faculty of Mechanical Engineering, Ho Chi Minh City University of Technology and Education, Ho Chi Minh City 71307, Vietnam; minhps@hcmute.edu.vn; 2 Faculty of Mechanical Engineering, Vinh Long University of Technology and Education, Vinh Long City 85110, Vietnam; lenhan@vlute.edu.vn

**Keywords:** deep drawing process, camera cover, formability, thickness, stress

## Abstract

The formability of the drawn part in the deep drawing process depends not only on the material properties, but also on the equipment used, metal flow control and tool parameters. The most common defects can be the thickening, stretching and splitting. However, the optimization of tools including the die and punch parameters leads to a reduction of the defects and improves the quality of the products. In this paper, the formability of the camera cover by aluminum alloy A1050 in the deep drawing process was examined relating to the tool geometry parameters based on numerical and experimental analyses. The results showed that the thickness was the smallest and the stress was the highest at one of the bottom corners where the biaxial stretching was the predominant mode of deformation. The problems of the thickening at the flange area, the stretching at the side wall and the splitting at the bottom corners could be prevented when the tool parameters were optimized that related to the thickness and stress. It was clear that the optimal thickness distribution of the camera cover was obtained by the design of tools with the best values—with the die edge radius 10 times, the pocket radius on the bottom of the die 5 times, and the punch nose radius 2.5 times the sheet thickness. Additionally, the quality of the camera cover was improved with a maximum thinning of 25% experimentally, and it was within the suggested maximum allowable thickness reduction of 45% for various industrial applications after optimizing the tool geometry parameters in the deep drawing process.

## 1. Introduction

Deep drawing is one of the most common sheet metal forming processes that are widely used for production of parts in automotive, aerospace, mobile phone and camera cover [1,2,3,4]. The camera cover is a complex 3D-part with many corners and is difficult to form in the deep drawing process. The tool geometry parameters that affect the success or failure of a deep drawing operation are the radius of the punch, radius of the die and clearance between the punch and die. In addition, the defects of the drawn part can be the thickening, stretching and splitting. The splitting often occurs at the corners of the drawn part, due to high tensile stresses that cause the excessive thinning of the sheet metal [5,6,7,8,9].

Many recent studies on deep drawing techniques have been used to prevent defects and improve the formability of the drawn part. Mark Colgan and John Monaghan [10] studied on the initial stages of a combined experimental and finite element analysis of a deep drawing process. The results reported that the punch/die radii had the greatest effect on thickness of the deformed steel cups compared to blank-holder force or friction. The smaller was the punch/die radii, the greater was the punch force and the shorter was the final draw. Additionally, the type of lubrication was very much affecting the force on the punch. Although the authors concluded that the die radii were noted as a prominent factor, they did not provide enough substance to account for other factors affecting the deep drawing process with complex 3D-parts. Duc-Toan Nguyen et al. [11] predicted the fracture of an AZ31B magnesium alloy sheet at warm temperatures and improved the press formability of the camera cover. The forming with temperature of 300 °C, blank holding force of 35 kN and friction coefficient of 0.03 was predicted to lead to better reliability than that of the original test sample. However, this method was costly for heating. The thermal treatments for deep drawing materials are commonly employed in an effort to improve formability or reduce residual stresses. Unai Ulibarri et al. [12] studied on the deep drawing process of Inconel 718 material with and without intermediate annealing thermal treatments based on the experimental and numerical simulation investigation. The behavior of the material in its as-received state and post-stretching thermal treatment state was analyzed and modeled. It was clear that the intermediate annealing process changed the microstructure beyond the relaxation of the dislocations that related to deformation. Additionally, although the roughness of the material increased during the pre-strain process, subsequent thermal treatments did not appear to affect the friction coefficient.

For the research progress of deep drawing of aluminum alloys, Paul Wood et al. [13] studied the effects of friction and back pressure on the formability of superplastically formed aluminum alloy AA7475 at a temperature of 517 °C. The results showed that back pressure had a significantly greater effect than friction in enhancing the formability of the sheet alloy material. In addition, a higher level of back pressure reduced the rate of growth of voids with strain in the material, thereby allowing greater thickness strain in the formed box. Muammer Gavas [14] applied a multi-point blank holder to increase the drawability of aluminum A1050 sheet. It concluded that the cup height with the multi-point blank holder was improved by 3.27 mm more than the normal blank holder one. Additionally, the improvement ratio of cup height was 6.13%. However, using a multi-point blank holder might not be the best method to obtain the highest limiting drawing rate (LDR), but it could increase the LDR substantially and be used for special purposes. In another study related to the deep drawing process of the cup by aluminum alloy material AA-1050, the author [15] examined the tensile strength and uniform elongation of this material shaped with the multi-stage for the cup of 50 mm in diameter and 100 mm in height. It was examined that a 4-stage drawing operation is needed in order to be able to obtain a cup with the aimed sizes. The experimental results showed that the material heaping occurred in the flange part, the material flowing was the most intensive in the wall region that remained under the blank holder at the start of drawing and deformed and formed the wall of the cup on its vertical axis at the end of the drawing. Additionally, it was observed that the tensile strength increased, and the ductility decreased at every stage of drawing. In this research, the tensile strength increased by 40% and the ductility decreased by 81% after 4- stages of drawing. For deep drawing of square cup-shaped parts with many edges and corners, Bharatkumar Modi and Diga Ravi Kumar [16] studied the effect of process parameters on formability of square cups by hydroforming from aluminum alloy AA5182 using both numerical simulation and experimental work. The process parameters were peak pressure, pressure path, and blank holding force for optimizing the formability that the criteria were the minimum thickness at the bottom corners and the minimum corner radius in the formed square cups. The results showed that the peak pressure was the most significant parameter affecting formability of square cups. With high peak pressure in the deep drawing process, the failure was at one of the bottom corners where the biaxial stretching was predominant mode of deformation and the thinning in this area was found to be 23–24% at failure. Robert Boissiere et al. [17] examined the effects of punch shapes on the deep drawing limits in expansion. Two punch shapes including the flat punch and the spherical punch were considered with the sheet material of aluminium alloy 2024. In this study, the strains were measured on the surface of samples based on the numerical and experimental works. In the case of the flat punch, the higher strains were located on a crown surrounding the flat surface. Additionally, it displayed the flat zone that did not undergo any friction with the punch. For the spherical punch, the central zone was not the most deformed and the maximal strain zone was located within a crown separating the punch contact and the no-contact zone. The results confirmed that larger maximal strains in biaxial stretching could be obtained in the case of the spherical punch.

**Figure 1:**
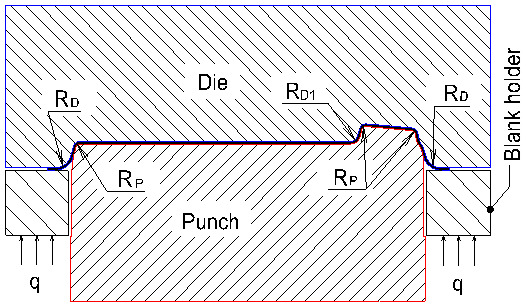
Geometry parameters of the die and punch; R_D_ is the die edge radius, R_D1_ is the pocket radius on the bottom of die, R_P_ is the punch nose radius and q is holder pressure.

In general, many factors can affect the quality of the drawn part such as material properties of sheet metal, thickness of the drawn part, blank holder pressure, press speed and tool geometry parameters [8,9,18,19]. The blank holder pressure and press speed are easy to adjust on the press machine. However, if the tool geometry parameters are changed, the punch and die must be re-manufactured. Therefore, optimization of tool geometry parameters is very necessary. For the deep drawing process of the camera cover, in this study, the splitting can occur at the corners. The tool geometry parameters need to be considered, including the die edge radius (R_D_), the pocket radius on the bottom of the die (R_D1_) and the punch nose radius (R_P_), as shown in Figure 1, to improve the formability of the camera cover without the fracture problem in the bottom corner areas (around R_P_ near R_D1_). The main purpose is to determine the optimal geometry parameters of the die and punch with the maximum thickness relating to the minimum stress of the von-Mises criterion of the drawn part for improving the quality of the camera cover.

## 2. Numerical Simulation and Experiment

### 2.1. Camera Cover Model

**Figure 2:**
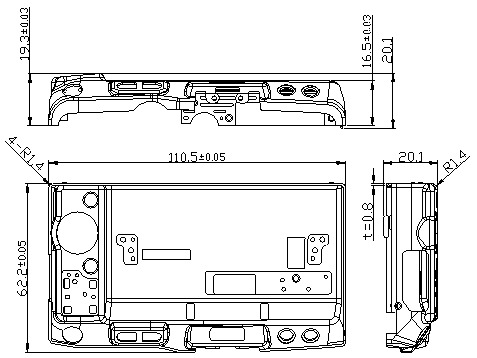
Camera cover model (unit: mm).

**Figure 3:**
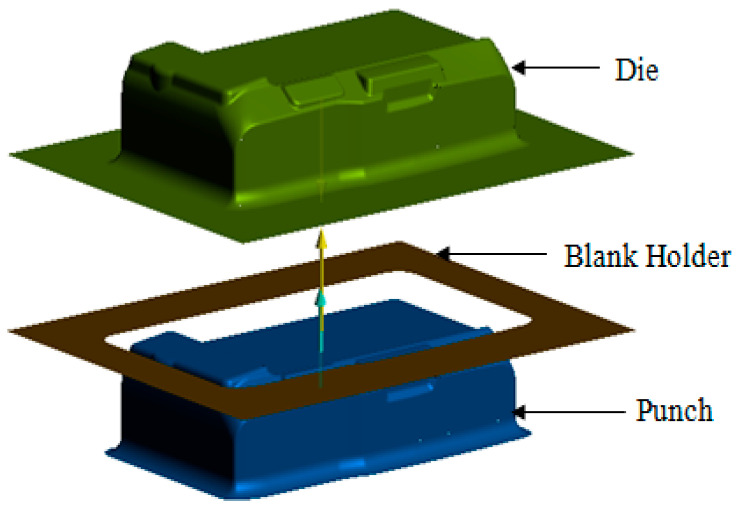
3D surface model of tools.

**Figure 4:**
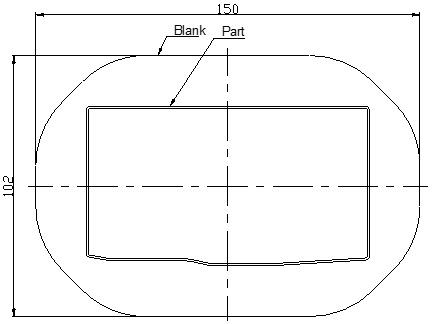
The shape of sheet blank (unit: mm).

The camera cover has the shape and dimensions as shown in Figure 2. It is a complex model with many corners and is difficult to form without defects such as scratching, wrinkling, puncturing and splitting [1,20,21]. For improving the formability of the camera cover, in this paper, the tool geometry parameters are optimized using numerical and experimental analyses. For simulating the deep drawing process on AutoForm software, the 3D surface models of tools including the die, punch and blank holder are designed as shown in Figure 3. The shape of the die is copied from the outside surface of the camera cover. The blank holder is designed based on the flat of die. The gap between the die and punch is selected as 0.85 mm, relating to the thickness of product [22]. The die, punch, and blank holder are modeled as rigid bodies and the sheet blank is modeled as the formable body. The sheet blank dimensions are determined based on the principle of balancing the volume of material before and after deformation (Figure 4). The blank sheet is meshed with the triangular element type and element numbers of 4000 for 13,460.6 mm^2^ [2,6,23].

### 2.2. Material Definition

Aluminum alloy materials are classified in the deep drawing materials group because they are easy to deform for making the complex 3D shapes [14,15], especially when used for camera covers. In this study, aluminum alloy A1050 is chosen with a sheet thickness of t = 0.8 mm and material properties according to the supplier as shown in Table 1. This material is a popular grade of aluminum alloy for the camera cover as moderate strength and formability are required [1,13,24]. 

### 2.3. Numerical and Experimental Conditions

The camera cover is formed by two stages. In the first stage, the shape of the bottom of the part is formed. In the second stage, the shape of the side wall of the part is formed and the radius adjusted at the corners. In this study, the servo press machine with a maximum load capacity of 110 tons and a maximum punch speed of 5 mm/s were used. The blank holder pressure is 4.5 N/mm^2^. The coefficient of friction between the holder and blank is 0.15 that relates to the surface quality of tools. All experiments were conducted at room temperature and at a relative humidity of 50 %.

Theoretically, the die edge radius (R_D_) should be as large as possible to permit full freedom of metal flow as it passes over the radius. However, if the die edge radius is too large, the metal will be released by the blank holder too soon and the wrinkling will result. The die edge radius has the direct effect on the formability of material, which may affect it in a positive or negative manner. A general rule to reduce the thinning is to design the die with the best radius of from 4 to 10 times the metal thickness. Since the value of the die edge radius depends on its relationship with the thickness of the camera cover, it is suggested that the value of R_D_ is considered from 3 to 8 mm. The pocket radius on the bottom of die (R_D1_) and the punch nose radius (R_P_) are further affected by the depth, edge radius, reduction percentage and material properties of the drawn part. The larger the punch nose radius, the lower the risk of splitting. However, the main constraint in the deep drawing die is the final geometry shape. In this study, the minimum radius of the final geometry shape of product is 0.8 mm. Therefore, the punch nose radius is chosen from 0.8 to 2 mm. If the punch nose radius is larger than 2 mm, the drawn part will crack at corners in the second stage (redrawing stage). The pocket radius on the bottom of the die is chosen from 2 to 4 mm. It is less than 4 mm because the depth of pocket is 5 mm. Finally, the chosen levels for the three parameters were shown in Table 2 [9,22].

**Figure 5:**
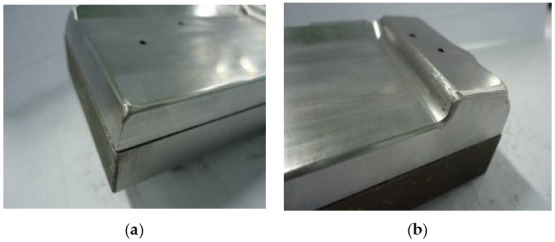
Two cases of the experimental punch. (**a**) Case I with R_p_ = 0.8 mm; (**b**) Case II with R_p_ = 2 mm.

**Figure 6:**
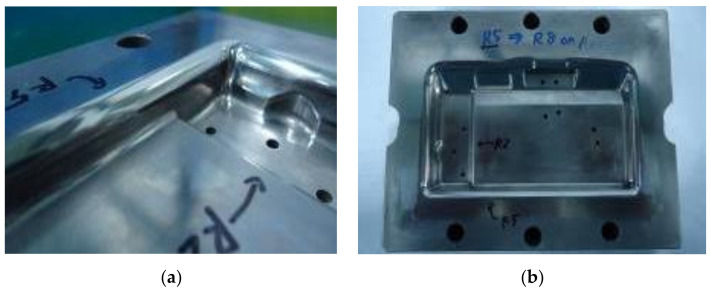
Two cases of the experimental die. (**a**) Case I with R_D_ = 5 mm and R_D1_ = 2 mm; (**b**) Case II with R_D_ = 8 mm and R_D1_ = 4 mm.

To compare the numerical and experimental results, the dies and punches are designed with two cases (Figure 5 and Figure 6). For the random selection (Case I), the die and punch with values of R_D_ = 5 mm, R_D1_ = 2 mm and R_P_ = 0.8 mm are performed as in Figure 5a and Figure 6a. For the optimal selection (Case II), the die and punch with values of R_D_ = 8 mm, R_D1_ = 4 mm and R_P_ = 2 mm, which refers to the numerical results, are performed as in Figure 5b and Figure 6b. In this study, a coordinate measuring machine (CMM, Belta-564-CNC+, Complete Precision Technology, New Taipei, Taiwan) is used to measure the experimental thickness with five samples for each evaluation to be compared with the numerical results. This is a three-dimensional measuring machine that measures the geometry of physical part by sensing discrete points on the surface of the part with a probe. 

## 3. Results and Discussion 

### 3.1. Thickness and Stress Distributions

**Figure 7:**
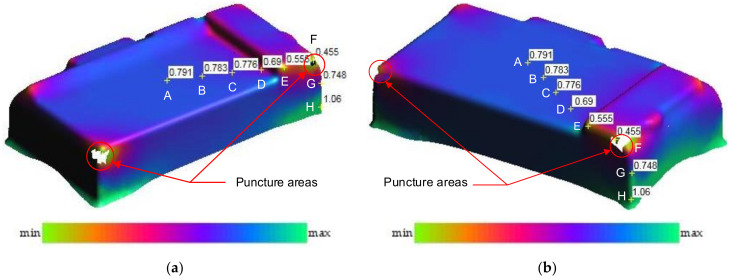
The numerical thickness distribution (unit: mm): (**a**) view 1; (**b**) view 2.

**Figure 8:**
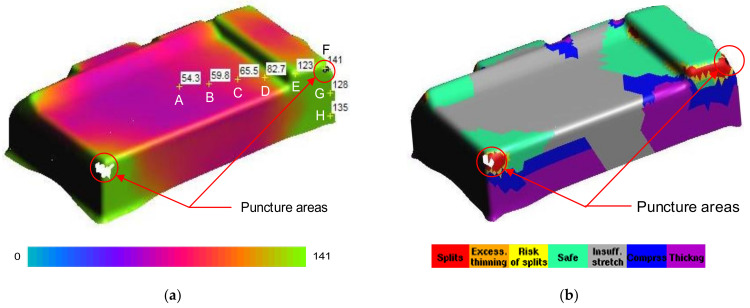
The numerical stress results: (**a**) stress distribution (unit: mm); (**b**) deforming area distribution.

Figure 7 and Figure 8 showed the thickness and stress distributions of the drawn part that occurred in the deep drawing process for Case I with R_D_ = 5 mm, R_D1_ = 2 mm and R_P_ = 0.8 mm. The numerical results in Figure 7 showed that the drawn part could be punctured on two corners as visible white areas due to too high thinning [16,17]. Especially, the deepest corner of the rectangular shaped pocket (point F) was easy to puncture, and was chosen to be examined in this study. Additionally, the thickening could occur in the flange area, the stretching occurred in both the bottom and the side wall areas, and the splitting occurred at the edge of bottom corners that related to the mode stress in the deep drawing process (Figure 8). The bottom area was subjected to the tensile stress that included points A, B, C, D, E and F. The thickness values decreased from 0.79 to 0.46 mm and the stress values increased from 54.3 to 141 MPa for from point A to point F, respectively. For the side wall with the vertical edge of the drawn part, it was subjected to both tensile and compressive stresses that included points G and H. The material was concentrated at point H causing the large values of thickness and stress. The thickness increased from 0.46 to 1.06 mm and the stress decreased from 141 to 135 MPa for from point F to point H, respectively. The thickness was the smallest as 0.46 mm and the stress was the highest as 141 MPa at the corner of the drawn part. It was clear that the camera cover might be fractured first at the bottom corners due to the high stress concentration [13,19,20]. These defects might occur because the geometry properties of the punch and die were reasonable. To prevent the fracture of the drawn part, it was necessary to optimize the geometry parameters of the die and punch for improving formability [9,18]. 

**Figure 9:**
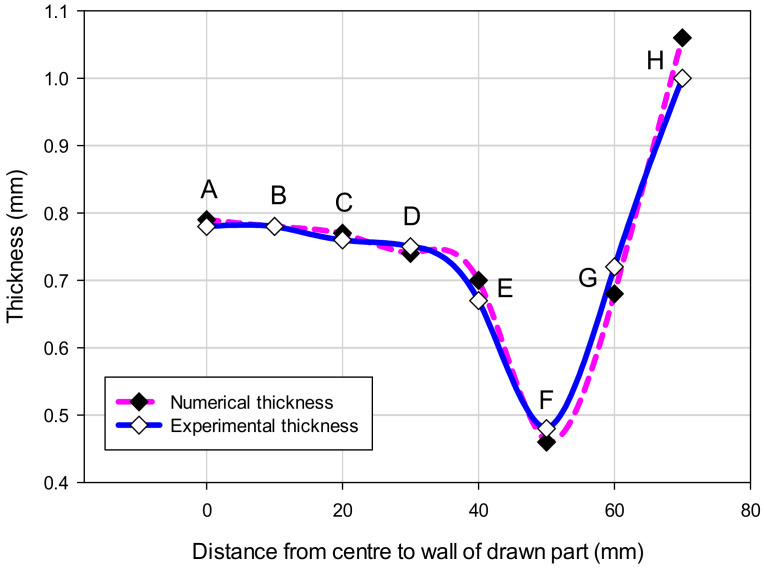
The thickness comparison of numerical and experimental results.

Figure 9 showed the thickness comparison of the numerical and experimental results for Case I with several points from the center to the wall of the drawn part, from point A to point H. For the experimental values, the mean thickness values of the five samples were measured by CMM at the same points with the numerical analyses. The results showed that the thickness distribution in the drawn part of the numerical results were a good agreement with that of experimental results. It was clear that the numerical analyses could be used to obtain the optimal geometry parameters of the tools with the maximum thickness relating to the minimum stress of the drawn part under the deep drawing process for improvement of the quality forming [2,6,23]. Especially, the result also showed that the thickness was non-uniformly distributed and it was the smallest at point F, which was the corner of the drawn part, and definitely affected the strength of the drawn part. Therefore, it needs to be considered for optimizing the thickness and the stress based on geometry parameters of the punch and die [5,25]. 

### 3.2. The Effect of Single Parameter on Thickness and Stress

**Figure 10:**
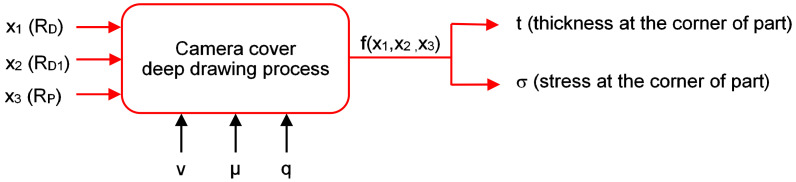
The schematic for the numerical and experimental analyses.

The effective parameters on different types of defects, which may appear in the drawn parts, can be divided into three main categories: material properties such as yield stress, work hardening coefficient and anisotropic coefficient; process parameters such as blank holder force, coefficient of friction and press speed; geometric parameters of tools such as punch radius, die radius and clearance. The optimum geometric parameters of tools lead to decrease in marking cost and trial cost. The important geometric parameters considered here are the die edge radius, the pocket radius on the bottom of die, and the punch nose radius. In this study, the dimension of the die depends on the outside surface of the part and the punch dimension is offset of 0.85 mm from the die surface. The analysis schematic of the experimental deep drawing process is designed as in Figure 10. The input parameters are the die edge radius, pocket radius on the bottom of die and the punch nose radius, the output values are the thickness and stress that are obtained from the numerical simulation results at the corners of the drawn part. In this study, the boundary conditions are the constant values of the press speed (v = 5 mm/s), friction coefficient (µ = 0.15) and blank holder pressure (q = 4.5 N/mm^2^). These parameters are chosen based on a combination of the sheet-metal forming experience and deep drawing process theory [9,22].

#### 3.2.1. The Die Edge Radius

Based on the analysis results in Section 3.1, the corners of the drawn part were the most thinning areas and should be examined both in thickness and stress through the variation of the die edge radius. Table 3 showed the numerical results of the thickness (t) and stress (σ) with different values of the die edge radius (R_D_) that obtained at the corners of the drawn part (point F) by AutoForm software. It was showed that the numerical results varied with increasing the die edge radius. This meant that the geometry of the die really affected the thinning of the drawn part that related to the stress in the deep drawing process.

Based on the simulation results in Table 3, the regression equations of thickness (1) and stress (2) as a function of die edge radius were determined by the least squares method as the following:

t = −0.00123(R_D_)^2^ + 0.0187R_D_ + 0.445 (1)

σ = 0.7286(R_D_)^2^ − 10.9886R_D_ + 181.7229 (2)

**Figure 11:**
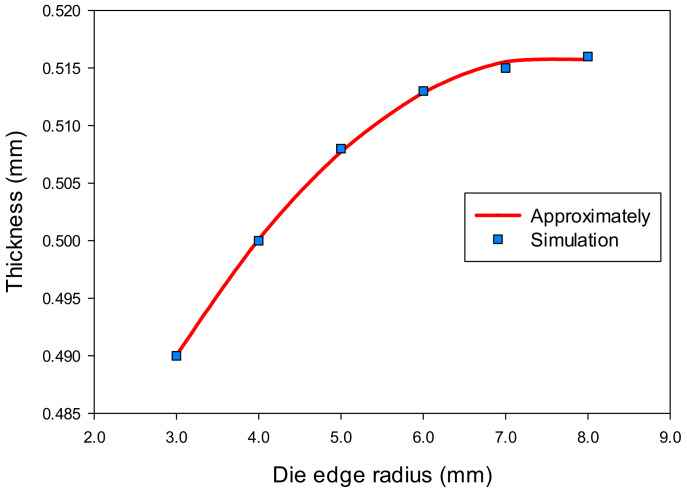
The effect of die edge radius on thickness.

**Figure 12:**
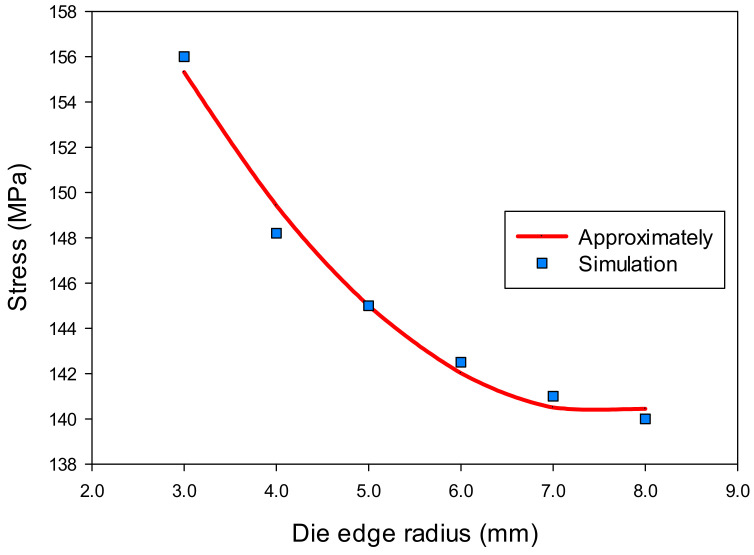
The effect of die edge radius on stress.

The effects of die edge radius on thickness and stress of the drawn part are described in Figure 11 and Figure 12. It clearly described that the changes on thickness and on stress were a consequence of an increasing die edge radius. When the die edge radius was too small, metal flow was difficult to move into cavity of die. When the die edge radius was larger than 8 mm (same as about 10 times of sheet thickness value), the thickness and was stress almost unchanged. When the die edge radius was too large, the wrinkling occurred in the flange of the drawn part. Normally, the die edge radius value was about 4–10 times of the blank thickness value [22,25].

#### 3.2.2. The Pocket Radius on the Bottom of Die

Table 4 shows the results of the thickness (t) and stress (σ) at the corner of the drawn part with the change of the pocket radius on the bottom of die (R_D1_). The thickness value increased from 0.516 to 0.526 mm and the stress value decreased from 143 to 138.5 MPa with the increasing the pocket radius on the bottom of the die from 2 to 4 mm, respectively. The regression equations were also found as Equation (3) for the thickness and Equation (4) for the stress of the drawn part as a function of the pocket radius on the bottom of die.

t = −0.00143(R_D1_)^2^ + 0.01357R_D1_ + 0.49466 (3)

σ = 0.8571(R_D1_)^2^ − 7.3429R_D1_ + 154.1857 (4)

**Figure 13:**
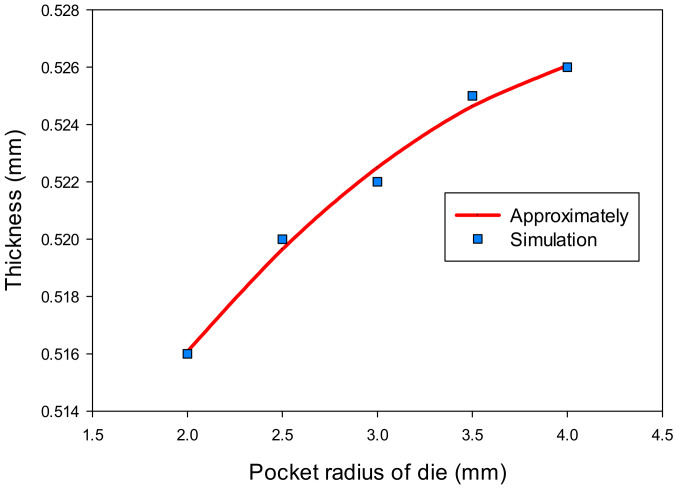
The effect of pocket radius of die on thickness.

**Figure 14:**
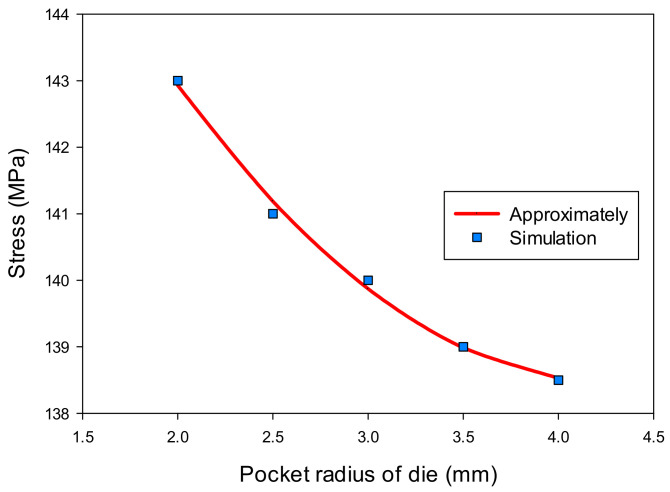
The effect of pocket radius of die on stress.

Moreover, the effects of the pocket radius on the bottom of the die on the thickness and stress of the drawn part are shown in Figure 13 and Figure 14. These figures clearly describe that the changes on thickness and on stress were a consequence of an increasing pocket radius of the die. The pocket radius of the drawn part was 2 mm. If the pocket radius was larger than 2 mm, the flow metal would move easily into the pocket. In this model, the pocket radius of die could not larger than 4 mm because the depth of the pocket was 5 mm.

#### 3.2.3. The Punch Nose Radius

Similarly, the variation results of the thickness (t) and stress (σ) of the drawn part at the corner with increasing the punch nose radius (R_P_) are shown in Table 5. The results show that the geometry design of the punch really affected the thickness and stress values that related to the formability of the drawn part in the deep drawing process. Based on these results, the regression equations were found depending on the variable of the punch radius as Equation (5) for the thickness and Equation (6) for the stress of the drawn part.

t = 0.03(R_P_)^2^ − 0.0315R_P_ + 0.5069 (5)

σ = 4.4643(R_P_)^2^ − 25.3571R_P_ + 165.5357 (6)

**Figure 15:**
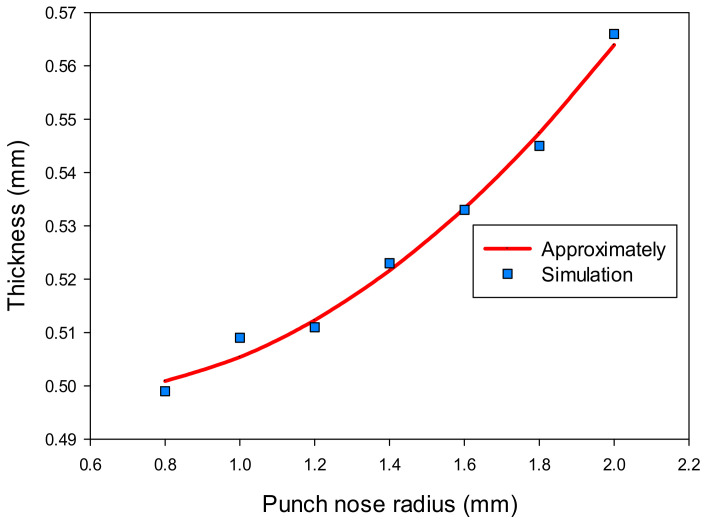
The effect of punch nose radius on thickness.

**Figure 16:**
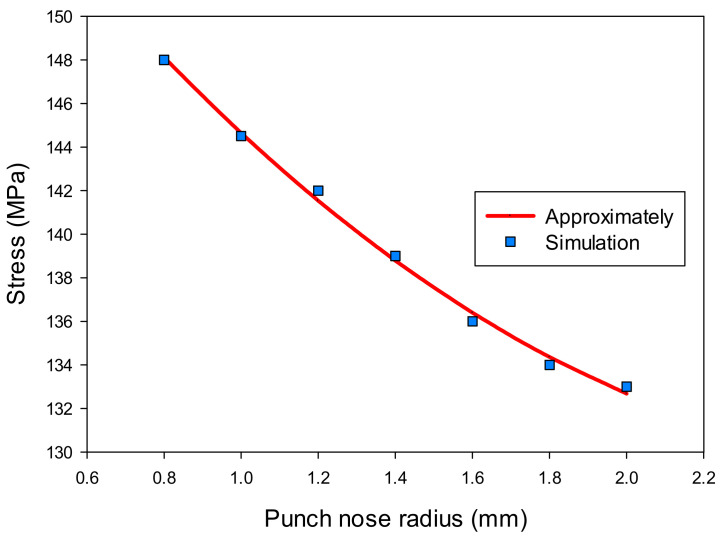
The effect of punch nose radius on stress.

The effects of the punch nose radius on the thickness and stress of the drawn part are shown in Figure 15 and Figure 16. The results showed that the thickness increased, and the stress decreased with varying the punch nose radius from 0.8 to 2 mm. It was clear that the punch nose radius should not be too large. It was difficult to form the drawn part in the second drawing stage if there were significant differences between the punch nose radius and radius of product in the same position.

### 3.3. Optimization of the Thickness and Stress of the Camera Cover

The formability of the drawn part was always based on multi parameters at the same time, it was necessary to consider multi parameters to find out the regression equation for optimizing the input parameters of tools [9,20]. The input parameters and coding variables were verified as in Table 6. The set of simulations was performed to investigate the optimal thickness of the camera cover, as shown in Table 7. According to statistical theory and numerical results, it was found the regression equation of optimization thickness (t) and stress (σ) that related to the die edge radius (R_D_), the pocket radius on the bottom of die (R_D1_) and the punch nose radius (R_P_) as the following:

t = 0.3098 + 0.0505R_D_ + 0.0045R_D1_ − 0.00455R_P_ + 0.0075R_D_R_P_ − 0.0042(R_D_)^2^ (7)

σ = 204.2011 − 14.6674R_D_ − 1.226R_D1_ − 0.0425R_P_ − 1.583R_D_R_P_ + 1.0956(R_D_)^2^ (8)

Equations (7) and (8) showed three parameters of die edge radius, pocket radius on the bottom of die and punch nose radius affecting the quality of the camera cover. Those equations could be used to control the deep drawing process. In there, the die edge radius was the largest effect, the die edge radius and punch nose radius interacted together and the pocket radius on the bottom of die was an independent factor that affected the thickness and stress of the drawn part. Based on these equations, the optimal geometry parameters of tools for the maximum thickness and the minimum stress at corners could be found. The maximum thickness at the corner was t = 0.57 mm and the minimum stress at the corner was σ = 125.9 MPa with R_D_ = 8 mm, R_D1_ = 4 mm and R_P_ = 2 mm. These results were approximate values and might be similar to or slightly different from simulation results. The result of optimization problem showed that the quality of the drawn part was the best, with maximum values of die edge radius, pocket radius on the bottom of die and punch nose radius in relevant ranges of parameters.

### 3.4. The Comparison of Numerical and Experimental Results

**Figure 17:**
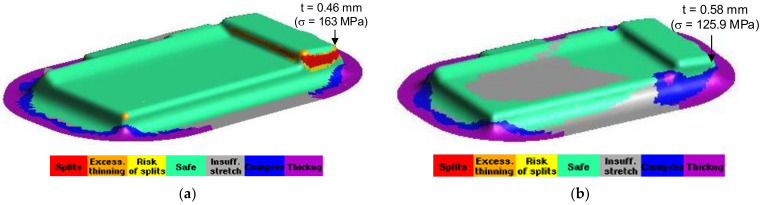
The numerical forming results. (**a**) Case I with R_D_ = 5 mm, R_D1_ = 2 mm and R_P_ = 0.8 mm; (**b**) Case II with R_D_ = 8 mm, R_D1_ = 4 mm and R_P_ = 2 mm.

**Figure 18:**
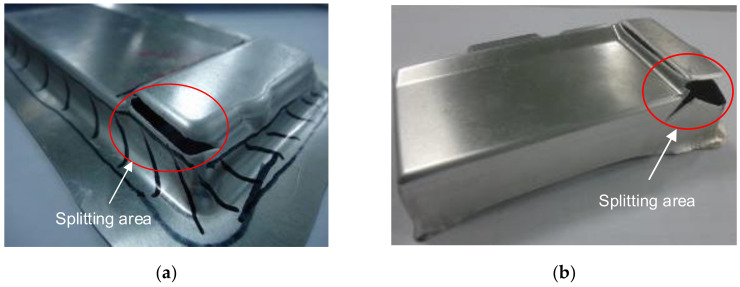
The experimental forming results for Case I. (**a**) The first stage; (**b**) the second stage.

**Figure 19:**
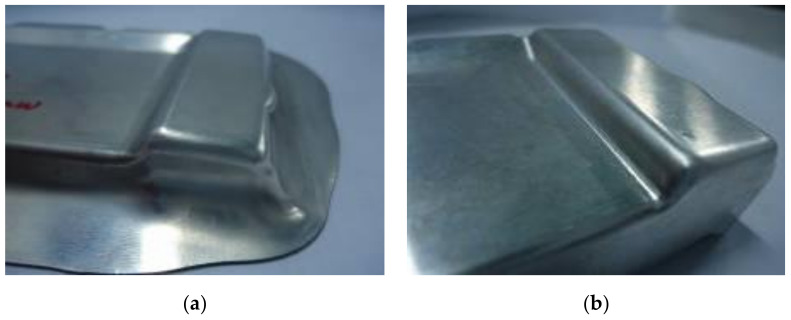
The experimental forming results for Case II. (**a**) The first stage; (**b**) the second stage.

For the comparison of the numerical and experimental results, several numerical and experimental works were conducted for Case I with R_D_ = 5 mm, R_D1_ = 2 mm and R_P_ = 0.8 mm, and for Case II with R_D_ = 8 mm, R_D1_ = 4 mm and R_P_ = 2 mm. Figure 17a,b showed the numerical forming results for Case I and Case II, respectively. The results showed that the quality of the drawn part was improved with a better thickness distribution when being performed by the optimal geometry parameters. For Case I, the thickness at the corner of the drawn part was 0.46 mm. In this case, the drawn part was split when it was performed by the tools with the random geometry parameters. In general, in the experimental tests the initial puncture appeared at one of the bottom corners which had the high biaxial stretching as well as the high stress concentration. Then, the spitting defect extended along the edge of bottom pocket to another area (around the edge between two adjacent corners R_P_, Figure 1) rather than occurring simultaneously at another corner. This phenomenon was seen from the camera cover test as shown in Figure 18. The numerical tests, which were approximate results, showed that the damage could appear at several corners of the drawn part (including the corner on the opposite side at R_P_, Figure 1), as shown in Figure 17a. However, both the numerical and experimental results showed that the most thinning area was around the bottom corners, as well as showing that the thickness distribution of both results were almost the same (Figure 9). For Case II, as the optimal selection, the numerical thickness value of the drawn part at the corner was 0.58 mm. Although this value was slightly different from the regression equation of optimization thickness (t = 0.57 mm), it was acceptable in the approximate numerical method. Thus, optimizing the die and punch parameters had improved the formability of the camera cover in the deep drawing process and this was proved experimentally, as shown in Figure 19.

Table 8 describes the thickness values obtained at the corners of the camera cover parts by simulation and experiment for Case I and Case II. The experimental thickness results were the mean values of five samples that were measured by CMM at the points corresponding to the numerical results. The thickness values between the five experiments were almost the same. However, the experimental stress values were not considered and do not appear in this table, because the stress measurements at the corners of the drawn part during the deep drawing process were too difficult without modern devices. It could be one of aspects to analyze in future works. Therefore, the stress results were only obtained from simulation results with values of 163 and 125.9 MPa for Case I and Case II, respectively. In addition, in production practice, measuring the thickness of the drawn part was easy to do with a precision machine of CMM and could be the target to control the deep drawing process. Table 8 shows that the thickness values between simulation and experiment were slightly different for both Cases I and II with 4.2% and 3.3%, respectively. Those small differences were inevitable for the numerical simulation method, and the experimental conditions might not be as stable as in the simulation. In general, the results between simulation and experiment were quite similar (Figure 9). The quality of the drawn part was improved when it was performed by the optimal geometry parameters. For Case I, the thinning at corners of the drawn part was 40% experimentally. In this case, the drawn part was fractured when it was performed by the tools with the random geometry parameters (Figure 17a and Figure 18). However, for Case II, the maximum thinning was only 25 % at corners of the drawn part experimentally. It was within the suggested maximum allowable thickness reduction of 45% for various industrial applications [25,26]. Thus, optimizing the die and punch parameters had improved the formability of the camera cover in the deep drawing process (Figure 17b and Figure 19). 

**Figure 20:**
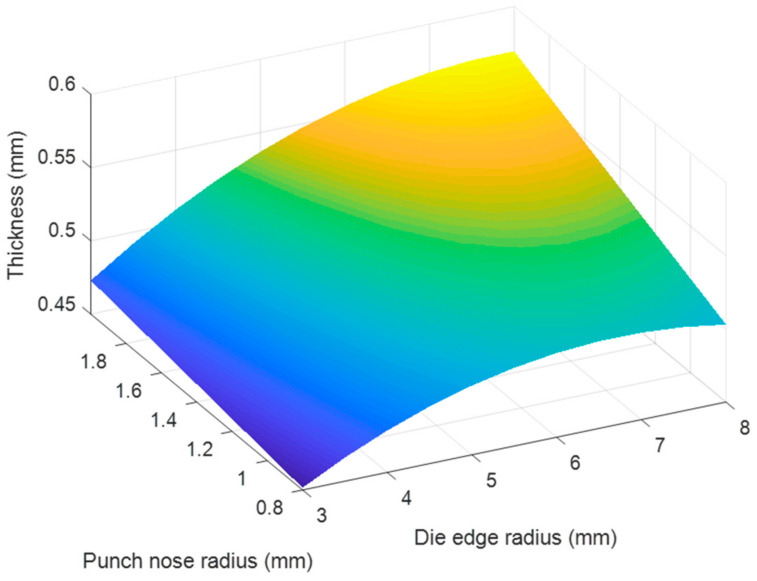
The effect of punch nose radius and die edge radius on thickness.

**Figure 21:**
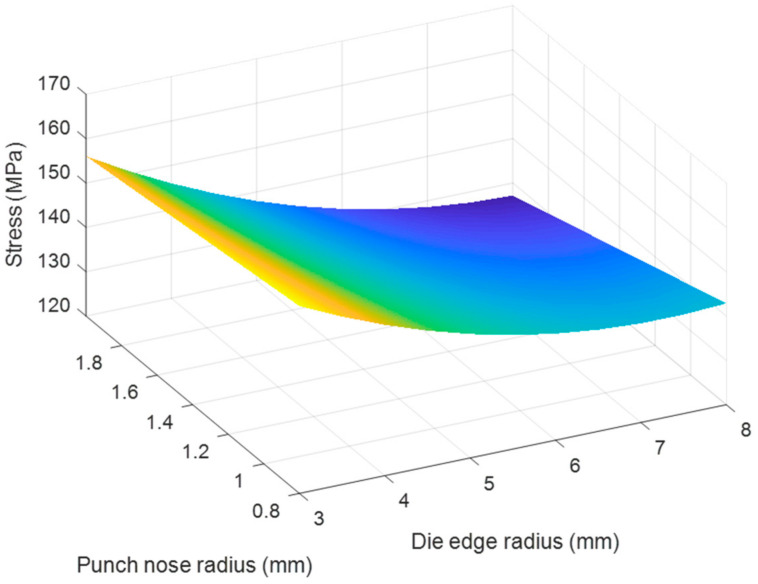
The effect of punch nose radius and die edge radius on stress.

**Figure 22:**
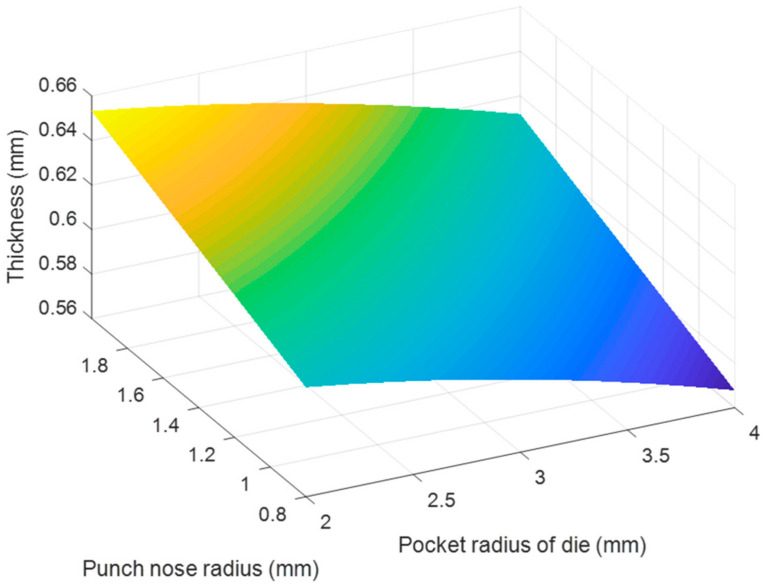
The effect of punch nose radius and pocket radius of die on thickness.

**Figure 23:**
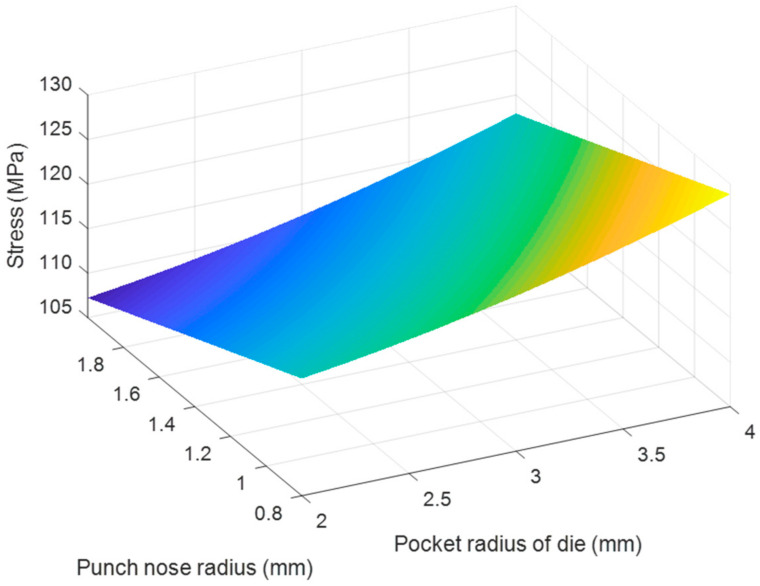
The effect of punch nose radius and pocket radius of die on stress.

**Figure 24:**
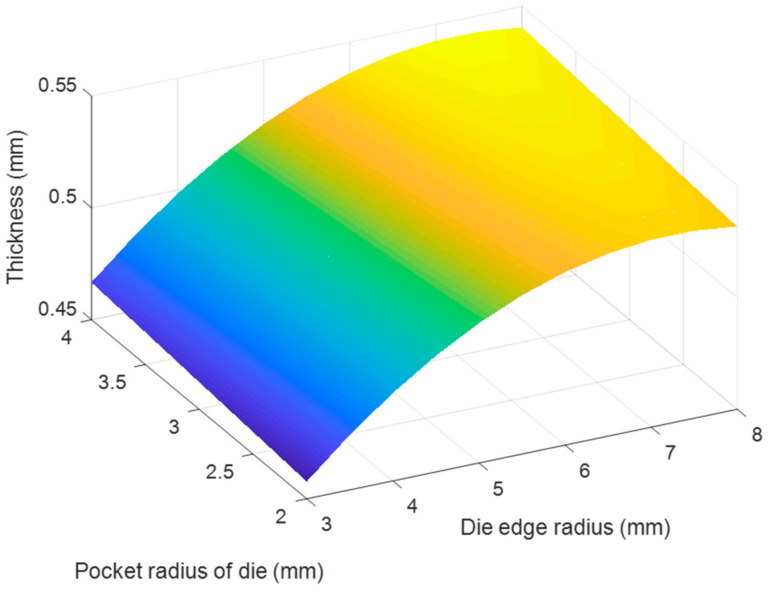
The effect of pocket radius of die and die edge radius on thickness.

**Figure 25:**
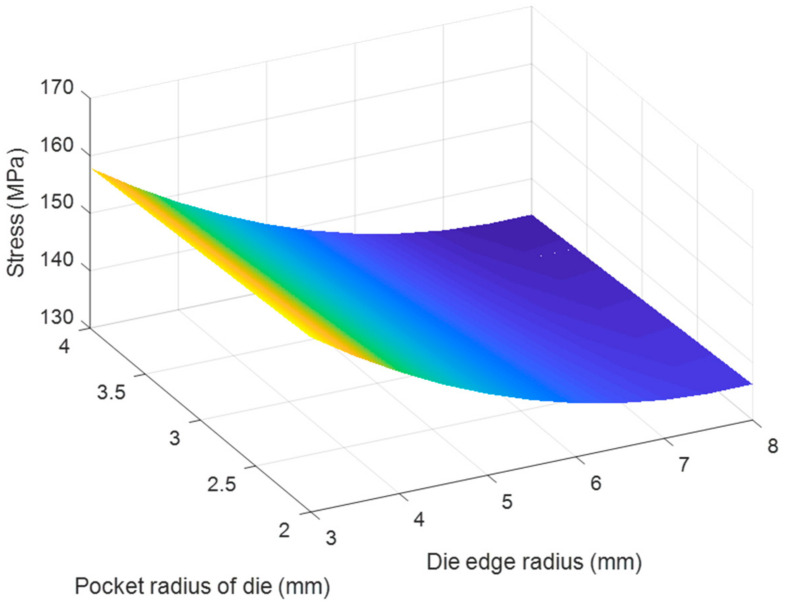
The effect of pocket radius of die and die edge radius on stress.

Based on the regression Equations (7) and (8), the effects of each pair of tool geometry parameters on the thickness and stress of the camera cover were considered with using MATLAB program and resulted in Figure 20, Figure 21, Figure 22, Figure 23, Figure 24 and Figure 25. The results showed that the changes on the thickness and on stress were the consequence of varying tool geometry parameters. It was observed that lower thinning was obtained at the bottom corners due to higher values of die edge radius, punch nose radius and pocket radius of die. In addition, the stress value of the drawn part decreased with increasing the die edges radius, pocket radius of die and punch nose radius. It was clear that the optimal thickness and stress of the drawn part could be predicted through the regression equations relating to parameters of the die edge radius, pocket radius on the bottom of die and punch nose radius [5,9].

## 4. Conclusions

Based upon experimental and numerical results, the following conclusions are drawn:

- The thickness distribution relating to the stress distribution of the drawn part could be found by numerical and experimental analyses. The results clearly described that the changes on the thickness and on the stress were the consequence of varying tool geometry parameters. It was observed that lower thinning was obtained at the corners due to higher values of the die edge radius, punch nose radius and pocket radius of die. The thickness was the smallest and the stress was the highest at one of the bottom corners where the biaxial stretching was predominant mode of deformation.
- The objective functions describing the effects of tool geometry parameters on the optimal thickness and stress of the drawn part were established by the statistic method. The optimization of tool geometry parameters including the die edge radius, the pocket radius on the bottom of die and punch nose radius had improved the formability of the camera cover with more uniform thickness distribution. In this study, a general rule to optimize the thickness distribution was to design the die and punch with the best values of the die edge radius of 10 times, the pocket radius on the bottom of die of 5 times and the punch nose radius of 2.5 times the sheet thickness.
- The thickening, wrinkling and splitting defects could also be prevented when the tool geometry parameters were optimized. It demonstrated that the quality of the camera cover was improved with a maximum thinning of 25 %, and it was within the suggested maximum allowable thickness reduction of 45% for various industrial applications.
- The regression equations obtained from this study can be referred for a wide range of products, especially for rectangular shaped products with many bottom corners, for improving the formability by the suitable selection of tool geometry parameters. However, the equation coefficients may different, depending on the materials, shape and dimension of the specific products.

## Figures and Tables

**Table 1:** Physical and mechanical properties of aluminum alloy A1050.

Elastic Modulus (GPa)	Yield Strength (MPa)	Tensile Strength (MPa)	Density (kg/m^3^)	Poisson’s Ratio	Hardness (HB)
71	95	150	2.71	0.3	34

**Table 2:** Tool parameters in the deep drawing process.

R_D_ (mm)	R_D1_ (mm)	R_p_ (mm)
3.0–8.0	2.0–4.0	0.8–2.0

**Table 3:** The thickness and stress results at point F with different values of die edge radius.

R_D_ (mm)	t (mm)	σ (MPa)
3.0	0.490	156.0
4.0	0.500	148.2
5.0	0.508	145.0
6.0	0.513	142.5
7.0	0.515	141.0
8.0	0.516	140.0

**Table 4:** The thickness and stress results at point F with different values of pocket radius on the bottom of die.

R_D1_ (mm)	t (mm)	σ (MPa)
2.0	0.516	143.0
2.5	0.520	141.0
3.0	0.522	140.0
3.5	0.525	139.0
4.0	0.526	138.5

**Table 5:** The thickness and stress results at point F with different values of punch radius.

R_P_ (mm)	t (mm)	σ (MPa)
0.8	0.499	147.0
1.0	0.509	145.0
1.2	0.511	144.0
1.4	0.523	140.0
1.6	0.533	137.0
1.8	0.545	134.0
2.0	0.566	133.0

**Table 6:** Input parameters and coding variables.

Input Parameters	Max	Mean	Min	Coding Variables	Max	Mean	Min
R_D_	8.0	5.5	3.0	X_1_	1	0	−1
R_D1_	4.0	3.0	2.0	X_2_	1	0	−1
R_P_	2.0	1.4	0.8	X_3_	1	0	−1

**Table 7:** The set of simulation.

No.	X_0_	X_1_	X_2_	X_3_	X_1_ X_2_	X_1_ X_3_	X_2_ X_3_	X_1_ X_2_ X_3_	X_1′_	X_2′_	X_3′_	t (mm)	σ (MPa)
1	+	−	−	−	+	+	+	−	0.27	0.27	0.27	0.451	163
2	+	+	−	−	−	−	+	+	0.27	0.27	0.27	0.498	144
3	+	−	+	−	−	+	−	+	0.27	0.27	0.27	0.463	160
4	+	+	+	−	+		−	−	0.27	0.27	0.27	0.506	143
5	+	−	−	+	+	−	−	+	0.27	0.27	0.27	0.472	157
6	+	+	−	+	−	+	−	−	0.27	0.27	0.27	0.559	130
7	+	−	+	+	−	−	+	−	0.27	0.27	0.27	0.479	154
8	+	+	+	+	+	+	+	+	0.27	0.27	0.27	0.572	126
9	+	0	0	0	0	0	0	0	−0.73	−0.73	−0.73	0.524	139
10	+	1.215	0	0	0	0	0	0	0.746	−0.73	−0.73	0.539	133
11	+	−1.215	0	0	0	0	0	0	0.746	−0.73	−0.73	0.427	167
12	+	0	1.215	0	0	0	0	0	−0.73	0.746	−0.73	0.529	139
13	+	0	−1.215	0	0	0	0	0	−0.73	0.746	−0.73	0.521	141
14	+	0	0	1.215	0	0	0	0	−0.73	−0.73	0.746	0.557	134
15	+	0	0	−1.215	0	0	0	0	−0.73	−0.73	0.746	0.494	148

+: corresponds to Max, −: corresponds to Min, X_0_ is an imaginary variable, X_1′_ = X_1_^2^ − 0.73, X_2′_ = X_2_^2^ − 0.73, X_3′_ = X_3_^2^ − 0.73.

**Table 8:** The numerical and experimental thickness values of the camera cover parts.

	R_D_ (mm)	R_D1_ (mm)	R_P_ (mm)	Simulation t (mm)	Experiment t (mm)
Case I (Random)	5.0	2.0	0.8	0.46	0.48
Case II (Optimum)	8.0	4.0	2.0	0.58	0.6

## Data Availability

The data used to support the findings of this study are available from the corresponding author upon request.

## Nomenclature

3D-part	Three-dimensional part
LDR	Limiting drawing rate
CMM	Coordinate measuring machine
R_D_	Die edge radius
R_D1_	Pocket radius on the bottom of die
R_P_	Punch nose radius
σ	Von-Mises stress
t	Thickness
v	Press speed
µ	Friction coefficient
q	Holder pressure

## References

[1] Goodarzi M., Kuboki T., Murata M. (2007). Effect of die corner radius on the formability and dimensional accuracy of tube shear bending. Int. J. Adv. Manuf. Technol..

[2] Ayari F., Bayraktar E. (2011). Parametric finite element analysis for a square cup deep drawing process. J. Achiev. Mater. Manuf..

[3] Najmeddin A., Abotaleb J. (2013). Theoretical and experimental analysis of deep drawing cylindrical cup. J. Miner. Mater. Charact. Eng..

[4] Saani S., Mostafa H., Abdessalem C. (2015). Single stage steel cup deep drawing analysis using finite element simulation. Int. J. Eng. Res..

[5] Reddy A.C.S., Rajesham S., Reddy P.R., Kumar T.P., Goverdhan J. (2015). An experimental study on effect of process parameters in deep drawing using Taguchi technique. Int. J. Eng. Sci. Technol..

[6] Djavanroodi F., Derogar A. (2010). Experimental and numerical evaluation of forming limit diagram for Ti6Al4V titanium and Al6061-T6 aluminum alloys sheets. Mater. Des..

[7] Younis K.M., Jaber A.S. (2011). Experimental and theoretical study of square deep drawing. J. Eng. Technol..

[8] Hamed K. (2014). Studying the effect of sharpen matrix radius on deep drawing operation. JLS.

[9] Zein H., El-Sherbiny M., Abd-Rabou M., El-Shazly M. (2013). Effect of die design parameters on thinning of sheet metal in the deep drawing process. Am. J. Mech. Eng..

[10] Colgan M., Monaghan J. (2003). Deep Drawing Process: Analysis and Experiment. J. Mater. Process. Technol..

[11] Nguyen D.T., Kim Y.S., Jung D.W. (2012). Coupled thermomechanical finite element analysis to improve press formability for camera shape using AZ31B magnesium alloy sheet. Met. Mater. Int..

[12] Ulibarri U., Galdos L., De Argandoña E.S., Mendiguren J. (2020). Experimental and numerical simulation investigation on deep drawing process of inconel 718 with and without intermediate annealing thermal treatments. Appl. Sci..

[13] Paul W., Muhammad J.Q., Andrzej R. (2011). Effect of friction and back pressure on the formability of superplastically formed aluminium alloy sheet. Key Eng. Mater..

[14] Gavas M. (2006). Increasing the deep drawability of Al-1050 Aluminum sheet using multi-point blank holder. METABK.

[15] Karali M. (2011). Examination of the strength and ductility of AA-1050 material shaped with the multi-stage deep drawing method. Arch. Metall. Mater..

[16] Modi B., Kumar D.R. (2019). Optimization of process parameters to enhance formability of AA 5182 alloy in deep drawing of square cups by hydroforming. J. Mech. Sci. Technol..

[17] Boissiere R., Vacher P., Blandin J.J. (2010). Influence of the punch geometry and sample size on the deep-drawing limits in expansion of an aluminium alloy. Int. J. Mater. Form..

[18] Lin B.T., Yang C.Y. (2017). Applying the Taguchi method to determine the influences of a microridge punch design on the deep drawing. Int. J. Adv. Manuf. Technol..

[19] Nguyen D.T., Park J.G., Kim Y.S. (2010). Ductile Fracture Prediction in Rotational Incremental Forming for Magnesium Alloy Sheets Using Combined Kinematic/Isotropic Hardening Model. Metall. Mater. Trans. A.

[20] Venkateswarlu G., Davidson M.J., Tagore G.R.N. (2010). Influence of process parameters on the cup drawing of aluminium 7075 sheet. Int. J. Eng. Sci. Technol..

[21] Li Y., Luo M., Gerlach J., Wierzbicki T. (2010). Prediction of shear-induced fracture in sheet metal forming. J. Mater. Process. Technol..

[22] Ivana S. (2006). Handbook of Die Design.

[23] Pourkamali A., Shahabizadeh M., Babaee B. (2012). Finite element simulation of multi stage deep drawing processes & comparison with experimental results. World Acad. Sci. Eng. Technol..

[24] Rydz D., Stradomski G., Szarek A., Kubik K., Kordas P. (2020). The analysis of pressed cups producing possibilities from rolled bimetallic Al-1050 + Cu-M1E sheets. Materials.

[25] Sherbiny M.E., Zein H., Abd-Rabou M., Shazly M.E. (2014). Thinning and residual stresses of sheet metal in the deep drawing process. Mater. Des..

[26] Prasad K.S., Panda S.K., Kar S.K., Murty S.V.S.N., Sharma S.C. (2018). Prediction of fracture and deep drawing behavior of solution treated Inconel-718 sheets: Numerical modeling and experimental validation. Mater. Sci. Eng. A.

